# Supplementary material for: A Likelihood Approach for Real-Time Calibration of Stochastic Compartmental Epidemic Models
Source: PLoS Comput Biol. 2017 Jan 17;13(1):e1005257. doi: 10.1371/journal.pcbi.1005257 (PMC5240920; doi:10.1371/journal.pcbi.1005257)
Supplement: S1 File — (TAR.GZ) [file pcbi.1005257.s014.tar.gz › HSPH_Online-SI-Revision/EnKF07/n1-extreme/n1-extreme_PF2_EnKF-summary2.pdf]

peak -8	3w spec	3w cum	5w spec	5w cum	end	3w spec LOG	3w cum LOG	5w spec LOG	5w cum LOG	end LOG
mean	280.4	982.2	82.5	285.2	52.7	0.7	0.8	1.2	0.6	0.5
median	219.8	783.1	81.3	263.6	45.9	0.7	0.8	1.2	0.6	0.4
quantile .1	81.1	156.6	63.3	92.9	35.2	0.5	0.4	0.7	0.4	0.3
quantile .9	574.7	1904.4	97.	518.4	82.4	1.1	1.2	2.	0.4	1.
44	16	29	30	35						

peak -4	3w spec	3w cum	5w spec	5w cum	end	3w spec LOG	3w cum LOG	5w spec LOG	5w cum LOG	end LOG
mean	82.2	91.7	91.1	74.1	74.1	1.1	0.5	1.8	0.6	0.8
median	84.2	89.7	91.6	73.	75.7	1.	0.4	1.8	0.6	0.8
quantile .1	67.5	71.5	84.1	54.3	55.3	0.6	0.3	1.3	0.3	0.4
quantile .9	94.2	111.2	96.4	93.3	88.6	1.6	0.9	2.3	0.3	1.3
17	39	0	34	21						

peak	3w spec	3w cum	5w spec	5w cum	end	3w spec LOG	3w cum LOG	5w spec LOG	5w cum LOG	end LOG
mean	92.2	94.	94.7	90.5	87.7	1.5	0.8	1.9	0.9	1.
median	92.2	91.6	94.3	89.	87.9	1.5	0.8	1.8	0.9	1.
quantile .1	87.1	82.7	92.1	82.8	81.8	1.2	0.7	1.6	0.8	0.8
quantile .9	97.1	107.1	98.1	98.2	92.8	1.7	0.9	2.1	0.8	1.1
7	24	1	26	22						

peak +4	3w spec	3w cum	5w spec	5w cum	end	3w spec LOG	3w cum LOG	5w spec LOG	5w cum LOG	end LOG
mean	122.1	124.2	119.6	118.5	111.	1.2	0.6	1.5	0.7	0.7
median	107.	115.	101.8	109.5	100.7	1.1	0.6	1.5	0.7	0.8
quantile .1	93.7	90.6	92.5	90.4	90.1	0.7	0.4	1.	0.4	0.5
quantile .9	158.	168.2	130.7	155.6	136.5	1.6	0.8	1.9	0.4	1.
26	41	17	38	36						

peak +8	3w spec	3w cum	5w spec	5w cum	end	3w spec LOG	3w cum LOG	5w spec LOG	5w cum LOG	end LOG
mean	292.8	358.4	822.7	332.1	304.4	0.9	0.5	1.	0.5	0.5
median	184.	276.5	177.7	227.8	196.5	0.9	0.5	1.	0.5	0.5
quantile .1	96.6	143.1	96.	126.8	116.	0.6	0.3	0.7	0.3	0.4
quantile .9	683.5	685.2	844.	644.5	534.9	1.2	0.6	1.5	0.3	0.7
46	48	41	47	46	{}	{}	{}	{}	{}	{}
